# Immunodeficiency-Associated Lymphoid Hyperplasia As a Cause of Intussusception in a Case of Activated PI3K-δ Syndrome

**DOI:** 10.3389/fped.2017.00071

**Published:** 2017-04-19

**Authors:** Daniel Mettman, Isabelle Thiffault, Chitra Dinakar, Carol Saunders

**Affiliations:** ^1^Department of Pathology and Laboratory Medicine, University of Kansas School of Medicine, Kansas City, MO, USA; ^2^Center for Pediatric Genomic Medicine, Children’s Mercy Hospital, Kansas City, MO, USA; ^3^Department of Pathology and Laboratory Medicine, Children’s Mercy Hospitals, Kansas City, MO, USA; ^4^Stanford University School of Medicine, Stanford, CA, USA

**Keywords:** lymphoid hyperplasia, PIK3CD, sinopulmonary infections, primary immunodeficiency, B-cell lymphomas

## Abstract

Activated PI3K-δ syndrome refers to a recently described primary immunodeficiency syndrome consisting of recurrent sinopulmonary infections, lymphadenopathy, mucosal lymphoid aggregates, increased susceptibility to Epstein–Barr virus and cytomegalovirus, and increased incidence of B-cell lymphomas. Variants in *PIK3CD*, which encodes the phosphatidylinositol-4,5-bisphosphate 3-kinase catalytic subunit delta isoform, enhance membrane association and kinase activity, resulting in increased signal transduction through the PI3K–Akt pathway. Whole-exome sequencing revealed a pathogenic *PIK3CD* variant in a patient with history of immunologic impairment, recurrent sinopulmonary infections, and lymphoid hyperplasia presenting as intussusception. This case illustrates that while lymphoid hyperplasia secondary to immunodeficiency is most often unsurprising and non-threatening, it can present as an emergency-like intussusception.

## Introduction

Pathogenic variants in a number of genes cause primary immunodeficiencies (PIDs), which predispose to infections. One such PID, activated PI3K-δ syndrome (APDS), is associated with dominant gain-of-function variants in *PIK3CD* encoding p110δ, the catalytic subunit of phosphoinositide 3-kinase δ. This protein is selectively expressed in leukocytes and is critical for lymphocyte biology. In mammals, there are three classes of PI(3)K that are distinct in their mechanisms of regulation, substrate specificity, and structure. Among the class I PI(3)K molecules, only p110δ (OMIM: 602839) is restricted to leukocytes and has specialized functions in adaptive immunity (1–5). Various germline or *de novo*, heterozygous gain-of-function variants in the *PIK3CD* gene have been reported in patients presenting with sinopulmonary infections, lymphadenopathy, nodular lymphoid hyperplasia, and cytomegalovirus and/or Epstein–Barr virus viremia (6, 7). One such variant, E1021K, has been described in several affected individuals (6, 8–12). Here, we report a patient with p.E1021K with a unique presentation.

In 2009, a 6-year-old boy presented with abdominal pain, constipation, and encopresis. Imaging revealed intussusception that was only temporarily reduced with air enemas, necessitating an emergency laparotomy that revealed a causative ileocecal valve mass. On microscopic examination, the mass consisted of non-granulomatous hyperplastic lymphoid tissue that was determined to be non-neoplastic by immunohistochemistry and fluorescent *in situ* hybridization. Surgical resection resolved the symptoms, but in the context of the patient’s medical history the lymphoid hyperplasia could not simply be ascribed to infection. His birth and past medical history revealed that after an uneventful term gestation and delivery, his development was unremarkable until cessation of breastfeeding at 2 years of age, after which he began to experience recurrent episodes of otitis media and sinusitis. That year he developed pulmonary symptoms that required a hospitalization and which eventually led to the development of bronchiectasis. Throughout the following 3 years, he continued to experience upper respiratory infections and recurrent otitis media with hearing impairment secondary to tympanic membrane rupture for which he underwent bilateral myringotomy with tympanostomy tube placement, tonsillectomy, adenoidectomy, and sinus evacuation.

Pertinent negative testing included *Histoplasma* serology, total complement activity, sweat chloride testing for cystic fibrosis, granulocyte oxidative burst testing for chronic granulomatous disease, and a tracheal cilia biopsy for ciliary dyskinesia. Quantitative total immunoglobulin and immunoglobulin subclass assays were repeatedly within normal limits except for consistently elevated levels of IgM (Table 1). However, while he demonstrated adequate functional response to protein antigens, despite repeated vaccination with conjugate pneumococcal vaccine and the pneumococcal polysaccharide vaccines, his pneumococcal titers were suboptimal, indicating functional antibody deficiency. Flow cytometric quantitative analysis of lymphocytes revealed a reduced number of T cells and a persistently decreased CD4:CD8 ratio. He had a low normal number of B cells, mildly low number of memory B cells, and a proportionately mild decrease in class-switched memory B cell subset (Table 1), but without evidence of class-switch blockage. His T cell function assay demonstrated evidence of impairment with low response to mitogens, normal response to *Candida* antigen, and no response to tetanus antigen. Testing for toll-like receptor function and the mannan-binding lectin pathway was unremarkable. Replacement subcutaneous gammaglobulin therapy was started in light of his history of recurrent sinopulmonary infections causing significant morbidity, functional antibody deficiency, and diminished T cell numbers and function. He subsequently developed significant cervical and occipital lymphadenopathy that was non-tender and not associated with any systemic symptoms, suggestive of infectious etiology.

**Table 1:** **Immune evaluation of patient with PIK3CD-related disease**.

Parameters	Normal	2015	2014	2013	2012	2011
Abs lymph (μL)	1.50–4.90	1.09	0.94	0.82	0.54	1.17
WBC (μL)	4.50–14.50	–	–	–	6.92	4.26 L
Lymph %	95–100%	–	–	–	16	21.1
F lymph abs (mm^3^)	1,400–4,000	–	–	–	1,107 L	899 L
CD45 %	95–100%	96.05	99.83	98.31	98.68	99.9
T cells plus (CD2+) %	95–100%	76	–	–	80	86
T cells plus (CD2+) abs	1,100–3,800	829 L	–	–	886 L	773 L
T cells plus (CD3+) %	%	67	73	70	75	78
T cells plus (CD3+) abs	900–2,900	730 L	687 L	575 L	830 L	701 L
T helper cells %	95–100%	27	31	32	39	38
T helper cells abs	450–1,600	294 L	292 L	263 L	432 L	342 L
T cytotoxic cells %	%	37	39	34	32	36
T cytotoxic cells abs	300–1,000	403	367	279 L	354	324
Helper/cytotoxic ratio (CD4/CD8)	1.20–2.99	0.73 L	0.79 L	0.94 L	1.22	1.06 L
**T cell interpretation**						
Total B cells (CD19+) %	%	13	–	16	15	12
Total B cells (CD19+) abs	100–700	142	–	131	166	108
TBNK interpretation			–	–	–	–
Natural killer cells %	%	15	–	–	9	8
**Natural killer cells abs**	80–662	164	–	–	100	72 L
Activated T cells (CD25+) %	%	10	–	–	14	12
Activated T cells (CD25+) abs		109	–	–	155	108
Activated T cells (HLA-DR+) %	%	9	–	–	16	12
Activated T cells (HLA-DR+) abs		98	–	–	177	108
Natural killer T cells (CD3+/CD16, 56+) %	%	8	–	–	6	3
Natural killer T cells abs		87	–	–	66	27
**Serum immunoglobulins (mg/dL)**						
IgG	608–1,229		1,110	1,290	593	666
IgA	32–234	–	–	–	339	271
IgM	46–230	–	–	–	140	295
IgE	0–126	–	–	–	–	<2.0

*L, low*.

## Materials and Methods

### Microarray

Microarray-based comparative genomic hybridization was performed using a custom microarray chip design with ~180,000 oligonucleotide probes based on human genome sequence (hg18). Chips were designed by Baylor College of Medicine (Houston, TX, USA) and manufactured by Agilent Technologies (Santa Clara, CA, USA). Analysis software is based on human genome build 36 (hg18).

### Molecular Genetics

Exome sequencing was performed on a research basis for this patient and his healthy parents following informed consent. Genomic DNA was extracted from peripheral blood mononuclear cells using a Chemagen MSM1 robot (Perkin Elmer, Baesweiler, Germany). DNA was prepared utilizing the KAPA Biosystems library preparation kit (KAPA Biosystems, Woburn, MA, USA) followed by Illumina TruSeqExome enrichment (Illumina, San Diego, CA, USA). The proband’s sample was sequenced to 15.2 Gb for an average depth of ~120×. Bidirectional sequence was assembled and aligned to reference sequence (GRCh37/UCSC hg19). Alignment and variant calling was performed as previously published (13–15). Briefly, using custom-developed software, RUNES and VIKING, variants were filtered to 1% minor allele frequency, then prioritized by the American College of Medical Genetics categorization (16), OMIM (http://www.omim.org) identity, and phenotypic assessment. PCR to amplify exon 24 and flanking regions of *PIK3CD* for Sanger sequencing was carried out using primers specific to the region. Purified PCR products were sequenced in both directions using an ABI PRISM 3130 genetic analyzer and aligned to reference sequence NM___005026.3.

## Results

In light of a non-diagnostic immunologic work-up and short stature (Table 1), an array-CGH was performed, which was uninformative. The family was consented and enrolled in the CMH undiagnosed disease program, where trio-exome sequencing was performed on a research basis, revealing a *de novo* pathogenic variant in *PIK3CD*, c.3061G>A (p.E1021L). Comparison of rare single-nucleotide variants between samples confirmed parentage. During the extended period of time over which the work-up was carried out, he developed chronic cervical lymphadenopathy and a pneumonia requiring intravenous antibiotics. Since his APDS diagnosis, he has been stably managed on immunomodulatory agents, and has not subsequently experienced an abscess, deep-seated infection, osteomyelitis, chronic diarrhea, fungal infection, or most importantly, a malignancy.

## Discussion

As illustrated by this case, it can be difficult enough to ensure any immunodeficiency is the cause of clinical findings, especially a diagnosis as seemingly obscure as APDS. The clinical and laboratory findings can be non-specific and variable. When the diagnosis is suspected, it can be confirmed with genetic testing if a pathogenic variant is present. As such a rarely diagnosed condition, treatment is not well established but has been reported that rapamycin rectifies the T-cell defect *in vitro*, increases naïve T-cell levels *in vivo*, and improves the clinical course (7). Selective p110-delta inhibitors have been shown to be effective in individuals with relapsing indolent lymphomas (17) and have been suggested as potentially effective agents for individuals with APDS, but have yet to be demonstrated as such in clinical trials (6). As an entity that presents such challenge, greater understanding both in terms of clinical presentation and consideration is necessary for ensuring detection with optimal frequency and expeditiousness. Having been described only recently, so few times, and with phenotypic heterogeneity; refinement of the understanding of the clinical manifestations is necessary. This case confirms previously reported features while clarifying previous assumptions. As in previous cases, our patient experienced recurrent upper and lower respiratory infections, lymphadenopathy, mucosal lymphoid proliferation, a low CD4:CD8, elevated levels of IgM, and low antibody titers following vaccination. Unlike most other cases, however, total immunoglobulin levels and levels of subtypes were within normal limits. Additionally, while he was heterozygous for the E1021K variant, genetic testing of his parents confirmed the variant to be *de novo* as compared with the majority of cases who inherited a variant from a parent.

Despite relative overall insignificance by comparison with common diagnoses, publication of rare entities is important, so that they are kept in mind and detected when present. This is especially true for cases such as this one. In which failure to diagnose early may have detrimental long-term outcomes. While otitis media and sinusitis are common in children, recurrence and pneumonia are less so and as in this case they should prompt etiological investigation. Although bronchiectasis is a consistent feature of this syndrome, it is conceivable that earlier diagnosis and treatment could prevent its development by reducing chronic inflammation. If this boy had been treated prior to the ileocecal mass proliferating to such a size, the resected segment of bowel could have been saved. Even though currently this may not be considered a life-altering consequence by some, as with any medical intervention the effects cannot be absolutely appreciated. The appendectomy, for example, which has long been regarded as relatively inconsequential, is now understood to potentially harbor unintended consequences (18). Diagnosis and treatment were optimal given the current ability to detect and treat this condition. Its recent discovery and rarity have prevented more frequent consideration, routine diagnostic work-up, and the establishment of a recommended evidence-based treatment regimen. With this syndrome already known to carry an increased risk for B-cell lymphomas and *PIK3CD* becoming increasingly implicated in oncogenesis, this represents an entity for which early diagnosis is particularly imperative (19–21).

Additionally, this case is useful for its demonstration of two clinical features that should prompt consideration of this diagnosis. Most PIDs produce severe phenotypes that result in diagnosis during the first year of life. This boy’s genetic diagnosis was not made until he was 10 years of age, and the relative innocuousness prevented serious suspicion for the first 6 years of his life. Thus, this diagnosis should be higher on the differential when a PID is suspected in an older child.

## Concluding Remark

Finally, this case draws much needed attention to lymphoid hyperplasia. Any lymph node large enough to warrant a biopsy is likely at least reactive with lymphoid hyperplasia. Since lymph node biopsies are most commonly done to exclude malignancy, in the absence of malignancy lymph nodes are usually described as hyperplastic and the cause is often assumed to result from an indeterminable etiology. As such, lymphoid hyperplasia has become associated with benignity and reason for cessation of investigation. This case demonstrates how lymphoid hyperplasia can actually present as a medical emergency by serving as a lead point for intussusception. While the presentation is not usually so dramatic, caution is still in order, particularly for this diagnosis. APDS is associated with lymphoid hyperplasia, lymphadenopathy, and the development of B-cell lymphomas in the second or third decade of life. Therefore, the appearance of a lymphoma in the context of a history of repeated biopsies of hyperplastic nodes should not be unexpected. For this reason, it is imperative that one maintain a high degree of suspicion despite repeatedly negative biopsies.

## Ethics Statement

The Children’s Mercy Hospitals and Clinics ethical committee approved this case report after being reviewed and satisfied all the sections of the rules and regulations for the research at CMH including the consent from the parents. Written consent has been obtained from the parents/legal guardians for publication.

## Author Contributions

CS, CD, and DM carried out data collection and drafted the manuscript. CS, IT, and DM carried out editing of the manuscript and contributed to reviewing the data. All authors read and approved the final manuscript.

## Conflict of Interest Statement

The authors declare that the research was conducted in the absence of any commercial or financial relationships that could be construed as a potential conflict of interest.
